# Risk Factors for the Development of the Disease in Antiphospholipid Antibodies Carriers: A Long-term Follow-up Study

**DOI:** 10.1007/s12016-021-08862-5

**Published:** 2021-07-03

**Authors:** Rosalía Demetrio Pablo, Pedro Muñoz Cacho, Marcos López-Hoyos, Vanesa Calvo-Río, Leyre Riancho-Zarrabeitia, Víctor M. Martínez-Taboada

**Affiliations:** 1 Servicio de Oftalmología, Hospital Universitario Marqués de Valdecilla, Cantabria, Spain; 2 Gerencia de Atención Primaria IDIVAL, Servicio Cántabro de Salud, Cantabria, Spain; 3 Servicio de Inmunología, Hospital Universitario Marqués de Valdecilla, Cantabria, Spain; 4 Servicio de Reumatología, Hospital Universitario Marqués de Valdecilla IDIVAL, Cantabria, Spain; 5 Sección de Reumatología, Hospital de Sierrallana, Cantabria, Spain; 6 Facultad de Medicina, Universidad de Cantabria, Santander, Spain

**Keywords:** Antiphospholipid syndrome, Antiphospholipid antibodies, Thrombosis, Abortion, Primary prophylaxis

## Abstract

The natural history of antiphospholipid antibodies (aPL) carriers is not well-established. The objectives of the present study were (a) to study the probability of developing clinical criteria of antiphospholipid syndrome (APS), (b) to identify potential risk factors for developing thrombosis and/or obstetric complications, (c) to study the association between the antibody profile and development of APS, and (d) to determine the efficacy of primary prophylaxis. We retrospectively analyzed 138 subjects with positive aPL who did not fulfill clinical criteria for APS. The mean follow-up time was 138 ± 63.0 months. Thirteen patients (9.4%) developed thrombosis after an average period of 73.0 ± 48.0 months. Independent risk factors for thrombosis were smoking, hypertension, thrombocytopenia, and triple aPL positivity. Low-dose acetyl salicylic acid did not prevent thrombotic events. A total of 28 obstetric complications were detected in 92 pregnancies. During the follow-up, only two women developed obstetric APS. Prophylactic treatment in pregnant women was associated with a better outcome in the prevention of early abortions. The thrombosis rate in patients with positive aPL who do not meet diagnostic criteria for APS is 0.82/100 patients-year. Smoking, hypertension, thrombocytopenia, and the aPL profile are independent risk factors for the development of thrombosis in aPL carriers. Although the incidence of obstetric complications in this population is high (31.6%), only a few of them meet APS criteria. In these women, prophylactic treatment might be effective in preventing early abortions.

**Supplementary Information:**

The online version contains supplementary material available at 10.1007/s12016-021-08862-5.

## Introduction

Antiphospholipid syndrome (APS) is an acquired immune disorder defined by the presence of thrombosis and/or pregnancy morbidity along with positive antiphospholipid antibodies (aPL), such as anticardiolipin antibodies (aCL), anti beta 2 glycoprotein antibodies (AB2GPI), and lupus anticoagulant (LA) [1]. The APS diagnosis requires both clinical (thrombosis and/or obstetric complications) and analytical evidence (confirmed presence of aPL). This is stated in the Sapporo international consensus [1], and later revised in Sydney [2].

The estimated incidence of aPL carriers in the general population is 5% [3]. Recently, a higher incidence of antiphospholipid antibodies, especially antibodies not included in the classification criteria, has been described in the general population and related with subclinical arteriosclerosis [4].

The thrombosis rates in these patients are different according to the studied populations. Thrombosis rates of 3.8% have been reported in systemic lupus erythematosus (SLE) with positive aPL [5]. The annual incidence of thrombosis in patients with positive aPL antibodies but without history of thrombosis or obstetric manifestations is different between the reported studies, ranging from 0, in patients without associated disorders [6], to 1.3–2.8/100 patients-year, in studies that mix healthy population with SLE and other autoimmune diseases [7, 8]. Rates of 7.4/100 patient-years have been reported in women with recurrent abortions [5, 9]. Supplementary Table 1 summarizes the main studies published on this subject [5–8, 10–17].

Several predictive factors for thrombosis in patients with analytical but no clinical criteria for APS have been described. Among them, male gender [8], previous thrombosis [7, 8], smoking [10], hypertension [11], and SLE [18] are the most frequently cited. At the analytical level, LA has been the antibody most strongly associated with thrombosis [19]. Other authors have also found association with aCL IgG [7, 11] or with AB2GPI [8]. On the other hand, some authors have also found an increased risk of thrombosis in patients without clinical APS but with multiple positivity for the different aPL [10, 11].

There is no consensus regarding the aPL profile that better predicts the obstetric complications. Both LA and aCL have been reported in the literature by different authors. Opatrny et al. [20] reported a meta-analysis to measure the strength of association between recurrent fetal loss and the presence of aPL in women without autoimmune diseases. They concluded that LA was the antibody most strongly associated with recurrent fetal loss. Lockshin et al. [21], in a multicenter prospective PROMISSE study, found an increased risk of fetal loss in patients with thrombosis or SLE history and positive LA.

In relation to the primary prophylaxis of these patients, the debate is still opened. There is consensus to treat SLE patients with acetyl salicylic acid (ASA) at low doses to prevent arterial or venous thrombosis [22]. However, this is not clear in asymptomatic patients without associated diseases. Preventive treatment of obstetric events is supported by the use of ASA and heparin as secondary prophylaxis [23], but in primary prophylaxis, there is a lack of consensus.

In the present study, we aimed to analyze the incidence of thrombosis and obstetric complications in patients with positive serology without a clinical criterion of APS, the potential risk factors for developing clinical APS, and analyzing the role of the autoantibody profile and primary prophylaxis in the development of clinical manifestations of the disease.

## Material and Methods

### Selection of Patients

Retrospective data were collected from 138 patients without clinical criteria for APS but with confirmed positive serology (aCL and/or AB2GPI) at medium or high titers separated by a minimum of 12 weeks [2]. Patients were selected from the database of the Immunology Division of a tertiary hospital. A total of 1200 clinical records from aPL positive patients between 1999 and 2004 were reviewed. Exclusion criteria included patients with a clinical diagnosis of APS, absence of confirmation for serology, and low titers of aPL. LA data were available in 89 of these patients. The study was carried out in accordance with the Declaration of Helsinki and approved by the Ethical Committee of Clinical Research of Cantabria.

### Clinical Data

The clinical data of the patients were obtained through a retrospective review of the medical history according to a predefined protocol. Demographic data (age and sex), cardiovascular risk factors (hypertension, diabetes, dyslipidemia, smoking), associated diseases, presence of aPL (aCL IgG/M, AB2GPI IgG/M, LA), development of thrombosis, and/or obstetric disease, as well as the treatment received, were recorded.

### Determination of aPL

The immunology laboratory quantifies by commercial enzyme immunoassay in solid phase (ELISA) the presence of the following antibodies and isotypes of aPL: aCL of the IgG and IgM isotype and AB2GPI of the IgG and IgM isotype. The results are reported as quantitative and semiquantitative. Thus, the aCL are quantified in GPL (aCL IgG) or MPL (IgM aCL) according to the standard curve that is constructed in each test with 5 dilution points of the Harris/Sapporo standards. AB2GPI is quantified as U/ml. The criteria recommended by the International Society of Thrombosis and Hemostasis (ISTH) Scientific and Standardization Committee (ISTH) for the standardization of LA/APA were applied for the characterization of LA [24].

### Statistical Analysis

A database was generated using the SPSS statistics 20. The qualitative variables have been described using the percentages. In the quantitative variables, the data were adjusted to the normal distribution using the Kolmogorov–Smirnov test, using, depending on the case, the arithmetic mean and the standard deviation or the median and the interquartile range. In the hypothesis tests, we used chi-square test or Fisher exact test for differences between groups in qualitative variables. To assess differences in numerical variables between 2 groups, we used the Student *t* test or the Mann–Whitney test, according to the adjustment of the variables to the normal distribution. In order to quantify the strength of the association, the odds ratio (OR) was used with its 95% confidence interval. To estimate the independent effect of the different variables on categorical dichotomous variables, we used logistic regression with the “enter” method.

The incidence rate of first case on thrombosis was calculated and expressed as the number of thrombosis per 100 patients-year. Logistic regression analysis was used to evaluate the independent effect of triple positivity and thrombocytopenia on thrombosis risk, controlling for established cardiovascular risk factors (smoking, high blood pressure, and dyslipidemia). To evaluate the predictive capacity of the different models, we used the area under the curve (AUC), and we compared the curves by the DeLong test. In addition, a backward conditional model was developed.

A Kaplan–Meier survival analysis and the log-rank test were carried out to analyze the cumulative incidence of events in the different groups of patients. Because methodological heterogeneity between studies was anticipated, a random-effects (DerSimonian and Laird) model was used for pooling the data. The between-study heterogeneity was assessed by using *I*^2^ statistic. *I*^2^ values of 25%, 50%, and 75% were considered to represent low, moderate, and high heterogeneity, respectively [25]. The possibility of publication bias was assessed statistically by using Egger’s tests [26] and visual inspection of funnel plots. The influence of a potential publication bias was explored by using the Duval and Tweedie “trim and fill” procedure [27]. These methods were used for the purpose of sensitivity analysis [28].

The IBM SPSS 25 (IBM Corp. Released 2017. IBM SPSS Statistics for Windows, Version 25.0. Armonk, NY: IBM Corp.), MedCalc Statistical Software version 18.9 (MedCalc Software bvba, Ostend, Belgium; http://www.medcalc.org; 2018), and Comprehensive Meta-Analysis software version 2.2.064 (Biostat, Englewood, NJ) were used for statistical analyses.

## Results

We analyzed 138 patients with positive serology for aPL (ACL, AB2GPI). Only patients with confirmed positive serology (aCL and/or AB2GPI) at medium or high titers separated by a minimum of 12 weeks [2] were included. One hundred and nineteen were women, and 19 were men. The mean age of the total sample was 41.36 ± 16.1 years. We analyzed the incidence of vascular and obstetric events during a mean follow-up of 138 ± 63 months (Table 1).

### Analysis of Thrombotic Events in aPL Carriers

**Fig. 1:**
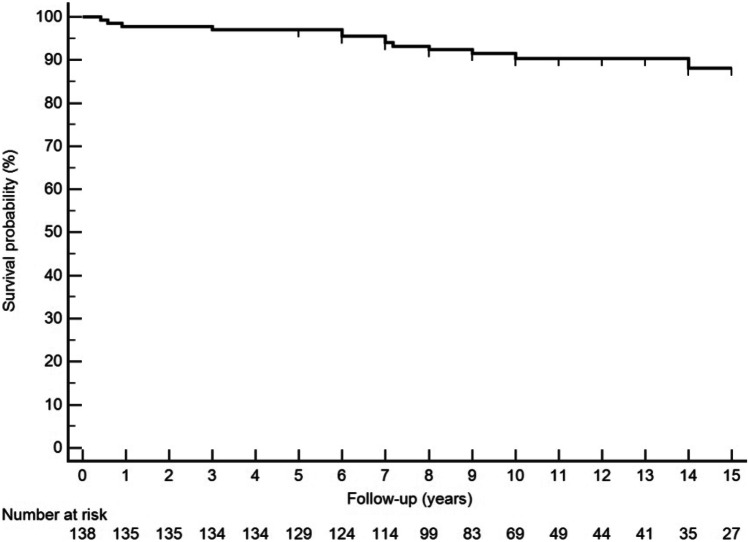
Thrombotic event-free survival curve. The figure shows the thrombosis-free survival during the follow-up. The incidence rate is 0.82 thrombosis per 100 patients-year (13 events/1591 total person-year). A relatively uniform incidence is seen within the first 10 years of follow-up. It should be noted that after the seventh year, the number of people under follow-up is <100

During the study period, thirteen patients (9.4%) developed thrombosis. The global thrombosis rate was 0.82/100 patients-year. The highest thrombosis rate was found in triple aPL-positive carriers (3.0/100 patients-year). The mean time to the thrombotic episode was 73.0 ± 48.0 months. Figure 1 shows the thrombosis-free survival during the follow-up. Table 2 shows the main demographic characteristics, associated diseases, and classic vascular risk factors of patients who developed thrombosis versus those who did not. Cardiovascular risk factors, especially smoking, hypertension, and dyslipidemia, were significantly more frequent in patients who developed thrombosis. As previously reported [28], 17 of the 138 (12%) patients in the present cohort had thrombocytopenia (platelet count ≤ 100,000/µl). The risk of developing thrombocytopenia was higher in smokers (OR 2.8; *p* = 0.044) and those with higher burden of aPL (OR 13.4; *p* < 0.001). During the follow-up, 5 patients with thrombocytopenia (29.4%) developed thrombosis (OR 5.9 [IC 95% 1.7–20.9]; *p* = 0.003). On the other hand, the presence triple aPL positivity (LA, aCL, AB2GPI) was associated with an increased risk for thrombosis (OR of 8.00, *p* = 0.027) (Table 2).

**Table 1:** Comparison between aPL carriers with and without thrombosis

	Thrombosis		
	No No. (%)	Yes No. (%)	*p*
Patients	125 (90.6)	13 (9.4)	
Female	109 (87.2)	10 (77)	0.306
Age, years(mean ± SD)	40.9 ± 16.3	45.5 ± 4.9	0.335
Associated diseases:			
SLE	23 (18.3)	4 (30.8)	0.285
CVRF			
Diabetes	2 (1.6)	0 (0)	0.646
Smokers	29 (23.2)	8 (61.5)	0.003
HBP	19 (15.3)	7 (53.8)	0.001
Dyslipidemia	7 (5.6)	5 (38.5)	0.001

*No.* number of patients, *SD* standard deviation, *p* statistical significance, *SLE* systemic lupus erythematosus, *CVRF* cardiovascular risk factors, *HBP* high blood pressure

**Table 2:** Frequency and odds ratio (OR) for thrombosis within the different serologic subgroups in aPL carriers. The OR were calculated taking as a reference category those patients positive only for aCL IgG

aPL serology	No. of patients	No. of thrombosis (%)	OR	*p*
aCL IgG (reference category)	38	2 (5.3)		
aCL Ig M	30	2 (6.7)	1.29	0.807
aCL IgG + aCL IgM	9	2 (22.2)	5.14	0.130
AB2GPI IgG	7	1 (14.3)	3.00	0.399
AB2GPI IgM	3	0 (0.0)	-	0.999
AB2GPI IgG + AB2GPI IgG	1	0 (0.0)	-	1.00
aCL + LA	13	1 (7.7)	1.50	0.749
aCL + AB2GPI	23	1 (4.3)	0.82	0.873
aCL + AB2GPI + LA	13	4 (30.8)	8.00	0.027
AB2GPI + LA	1	0 (0.0)	-	1.00
Total	138	13 (9.4)		

*No.* number, *aPL* antiphospholipid antibodies, *aCL* anticardiolipin antibodies, *AB2GPI* antibeta 2 glycoprotein antibodies, *LA* lupus anticoagulant

The next step was to evaluate the predictive capacity of different models to predict the development of thrombotic events in aPL carriers. Table 3 summarizes the OR, *p* value, and AUCs for the 7 models tested. Model 0 and the four-covariate models (M1, M2, and M3) have a very similar AUC, approximately 89%. Model 6, which includes smoking, HBP, and thrombocytopenia, is the only one with three variables that achieves an AUC of 89%. Although the difference in their respective AUCs was not significant (*p* = 0.311), thrombocytopenia predicts better the incidence of thrombosis than triple positivity (M5 vs. M6). The variables of M6 were selected using the backward conditional method with M0 (Supplementary Table 2).

**Table 3:** Multivariate analysis including classic cardiovascular risk factors, triple positivity, and thrombocytopenia

Model	OR (95% CI)	*p* value	AUC (%)
M0			89.7
Smoking	10.25 (2.18–48.24)	0.003	
HBP	8.74 (1.57–48.82)	0.014	
Dyslipidemia	2.87 (0.53–15.47)	0.220	
Triple positivity ^a^	2.31(0.28–19.08)	0.438	
Thrombocytopenia	4.09 (0.57–28.05)	0.159	
M1			89.9
Smoking	11.00 (2.38–50.53)	0.002	
HBP	6.92 (1.36–35.29)	0.020	
Dyslipidemia	3.63 (0.71–18.52)	0.121	
Triple positivity ^a^	5.20 (1.00–27.08)	0.050	
M2			
Smoking	10.12 (2.19–46.80)	0.003	89.1
HBP	9.77 (1.80–53.17)	0.008	
Dyslipidemia	2.73 (0.52–14.25)	0.233	
Thrombocytopenia	6.29 (1.27–31.02)	0.024	
M3			89.1
Smoking	11.69 (2.55–53.54)	0.002	
HBP	13.03 (2.66–63.72)	0.002	
Triple positivity^a^	2.11 (0.28–16.13)	0.473	
Thrombocytopenia	5.11 (0.78–33.35)	0.088	
M4			86.4
Smoking	11.25 (2.54–49.89)	0.001	
HBP	7.30 (1.50–35.46)	0.014	
Dyslipidemia	3.67 (0.77–17.57)	0.103	
M5			86.2
Smoking	12.38 (2.78–55.26)	0.001	
HBP	10.64 (2.37–47.71)	0.002	
Triple positivity^a^	5.30 (1.06–26.51)	0.042	
M6			89.2
Smoking	11.48 (2.54–51.87)	0.002	
HBP	14.19 (2.94–68.56)	0.001	
Thrombocytopenia	7.40 (1.55–35.20)	0.012	

*OR* odds ratio, *CI* confidence interval, *AUC* area under the curve, *HBP* high blood pressure

^a^Single or double versus triple

**Fig. 2:**
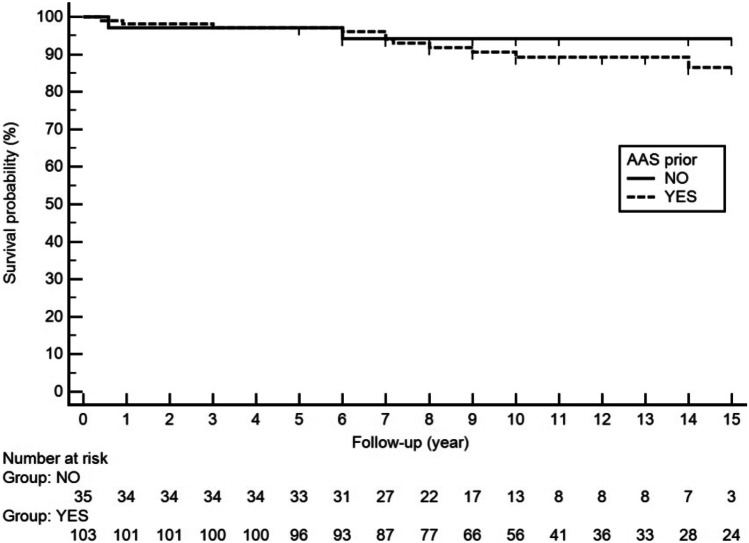
Kaplan–Meier thrombosis-free survival according to aspirin intake. There are no differences in the incidence of thrombosis during the first 7 years of follow-up between both groups. After this date, the differences are not significant (*p* = 0.393). AAS acetyl salicylic acid

Supplementary Table 3 shows the main characteristics of the 13 patients who developed thrombosis. Nine patients were taking ASA prior to the development of thrombosis. Of the 13 thrombotic events, 10 were arterial and 3 occurred in venous territory. Of the 138 patients, 103 received prophylaxis with ASA (100 mg/day). As shown in Fig. 2, aPL carriers on prophylactic treatment with low-dose ASA had a higher risk for thrombosis (hazard ratio 1.49 [95% CI 0.33–6.79]), although this difference was not statistically significant (*p* = 0.393).

### Obstetric Events in Women with Positive aPL

We analyzed 92 pregnancies in 39 women with positive aPL. LA data were available in 34 of the 39 women. Since many of the pregnancies occurred before aPL detection, we decided to carry out a second analysis of 38 pregnancies in 15 women who had a gestation after the positivity of the antibodies was detected. Only two patients evolved to obstetric APS (Supplementary Table 4) and none to thrombotic APS.

The results obtained with the two different analyses were as follows (Table 4):

1. Results of 92 pregnancies in 39 women before and after presenting positive serology for aPL. Overall, two-thirds of aPL carriers ended in a normal pregnancy. A total of 28 obstetric complications were detected in 26 of the 92 pregnancies. The mean age of pregnant women at delivery was 29.86 ± 5.8 years. The gestational age of the newborn presented an average of 38.2 ± 1.77 weeks, and the mean birth weight was 3108 ± 482.5 g. The mean Apgar Score was 8.66 ± 1.1. Only 8 out of the 39 women received treatment with ASA (100 mg/day), and ASA was associated with low weight molecular heparin (LWMH) in three of them. We did not obtain significant differences when we evaluated the influence of treatment on the total obstetric complications. However, prophylactic treatment was associated with a better outcome in the prevention of early abortions (< 10 weeks) with an OR of 0.12 (95% CI 0.02 to 0.95, *p* = 0.019).
2. Results of 38 pregnancies in 15 women after positive serology for aPL. A total of 12 obstetric complications were detected among the 38 pregnancies: 8 early abortions, one fetal loss, one preterm birth, and two IUGR. Six women received treatment with ASA (100 mg/day), together with LMWH in 3 of them. In this scenario, patients on prophylactic treatment showed a significant reduction on the total obstetric complications, with an OR of 0.15 (95% CI 0.02–0.85; *p* = 0.021). In agreement with the previous analysis, prophylactic treatment was also effective in the prevention of early abortions (< 10 weeks) with an OR of 0.40 (95% CI 0.26–0.62, *p* = 0.003)

**Table 4:** Description of obstetric complications in women after positive aPL versus pregnancies in women before and after positive aPL

	Pregnancies after aPL+ (No. = 38)		Pregnancies before and after aPL+ (No. = 92)	
Obstetric complication	No.	%	No.	%
Early pregnancy loss (< 10 weeks)	8	21	21	22.8
Fetal loss > 10 weeks	1	2.6	1	1.1
Preterm birth < 34 weeks	1	2.6	1	1.1
Intrauterine growth restriction	2	5.3	3	3.3
Preeclampsia	0	0	2	2.2
Normal pregnancy	26	68.4	64	69.6

*No.* number, *aPL* antiphospholipid antibodies

## Discussion

**Fig. 3:**
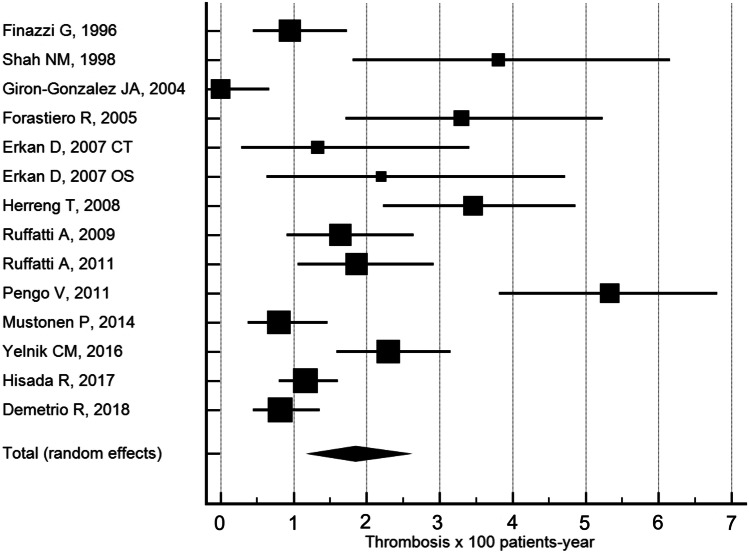
Forest plots of the incident rate of thrombosis per 100 patients-year using a random-effects analysis. The squares represent study-specific incident rate (the square sizes are proportional to the weight of each study in the overall estimate); the horizontal lines represent 95% confidence intervals (CI), and the diamond represents the overall incident rate estimate with 95% CI

The clinical course of patients with positive aPL serology who do not meet clinical criteria for APS has not been clearly established. Supplementary Table 1 reflects the variability in thrombosis rates reported by the different authors [5–8, 10–17]. As shown in Fig. 3, the thrombosis rate was highly variable depending on the different studies analyzed. Pooled random effect estimate was 1.7 thrombosis per 100 patients-year (IC 1.1–2.2). The present study showed a global thrombosis rate of 0.82/100 patients-year, which is located in the lower range when compared with other studies. However, when we analyzed only patients with triple positivity, the thrombosis rate increased up to 3.0/100 patients-year. In only two studies, Girón-González et al. [6] and Pengo et al. [11], the limits were not within the combined estimation (CI 1.1–2.2). As shown in Supplementary Fig. 1, and although it is not possible to find symmetry in the representation of the incidence rate, because incidences lower than 0% are not possible (shaded area); there is a lack of studies on the left side, corresponding to studies with low sample numbers and low incidence rates of thrombotic events. This means that there is a certain asymmetry with respect to the number of studies with an incidence of 0% or very close to it. The Egger’s test of the intercept was significant (*p* < 0.0001), suggesting again the existence of asymmetry. Using Duval and Tweedie “trim and fill” method, under the random effect model, the recomputed global estimation of the incident rate was 0.78 thrombosis per 100 patients-year (CI 0.24–1.32), a number that is clearly lower that the obtained directly from the studies (1.7 thrombosis per 100 patients-year), and very similar to the results of the present study (0.82 thrombosis per 100 patients-year). Nevertheless, the results of this metaanalysis should be taken with caution because of the high heterogeneity (*I*^2^ = 86.7%) of the different studies. Several factors might explain these diverging results—the follow-up time, the proportion of patients with associated diseases (especially SLE), the aPL profile, and the different therapeutic (primary prophylaxis) approaches. Furthermore, the influence of persistently positive serology, although relevant [30–32], has not been addressed in the majority of the studies.

Current evidence supports the theory that the presence of aPL is not the only risk factor necessary for developing thrombosis [33]. The coexistence of other factors, and more specifically classic cardiovascular risk factors, could act as triggers [10, 11]. In our study, smoking, hypertension, and dyslipidemia were identified as risk factors for thrombosis in this aPL carrier population. These findings support the concept that primary prophylaxis in aPL carriers should start by a proper treatment and correction of cardiovascular risk factors.

Thrombocytopenia is a non-criteria manifestation of APS. As recently reviewed [29], thrombocytopenia might be associated with a higher risk of thrombosis in aPL carriers, and also in patients with low aPL titters [17]. Although the difference in their respective AUCs was not significant (*p* = 0.311), thrombocytopenia better predicts the incidence of thrombosis than triple positivity (M5 vs. M6). These results might be explained because the linear association of thrombocytopenia with the burden of aPL [29].

Our study has the peculiarity of studying patients who were asymptomatic or who did not meet disease criteria, with a sample size of 138 patients and a mean follow-up of 138 ± 63 months. We obtained, as did other authors [10, 11], a statistically significant increase in risk among those who were positive for the three types of antibodies (LA, aCL, and AB2GPI), with an OR of 7.3 (95% CI 1.9–28.5, *p* = 0.004). The only published cohort without associated autoimmune diseases was published by Girón et al. [6], where none developed thrombosis probably because of the short follow-up (36 months). In our study, the mean time to the thrombotic event was 73.0 ± 48.0 months.

Regarding treatment, there is general agreement to treat APS that have had thrombosis or recurrent fetal loss to prevent new events [23]. However, it is unclear what to do in those patients with incidentally positive serology and no history of thrombosis or obstetric morbidity. It has only been studied more extensively in patients with SLE, where primary prophylaxis of these patients is accepted [22]. The different studies on primary thromboprophylaxis in patients with asymptomatic aPL positive patients are shown in Supplementary Table 1. There is only one clinical trial that addressed this issue which was carried out by Erkan et al. (APLASA study) [14], concluding that ASA did not protect against thrombosis. However, the limited number of patients, the short follow-up, and the low rate of thrombosis made it difficult to draw definitive conclusions. In the present study, ASA did not show a protective role in these patients (Fig. 2). In fact, aPL carriers treated with ASA showed a non-significant increase in the number of thrombotic events. This surprising finding might be related with the concept of confounding by indication. Furthermore, and as stated before, the lack of protective effect might be related to several factors, including the dose of ASA [34]. To clearly establish the role of primary prophylaxis in aPL carriers, it would be necessary to act on those modifiable risk factors, especially on all those related to cardiovascular risk. On the other hand, in those patients with associated independent risk factors, or with an elevated GAPSS score, primary prophylaxis should be considered. An international multicenter effort should address which is the best primary prophylaxis approach in aPL carriers stratified according to the risk of thrombosis.

The literature on obstetric outcome in patients with positive aPL who do not meet the clinical criteria of APS is scarce. Most published studies mix asymptomatic patients with patients with APS, SLE, and other autoimmune diseases. We have found few articles that analyze patients that meet the analytical but non-clinical criteria of APS. Chauleur et al. [35] analyzed the evolution of a second pregnancy in women with a previous abortion < 10 weeks and with positive aPL who did not meet APS criteria. LA and IgM aCL were associated with abortions. The aCL IgM and IgG were associated with late complications. The authors did not recommend screening for aPL in all patients with an early abortion because 70% of second pregnancies were successfully completed. Girón et al. [6] did not find any obstetric complications in a prospective study of a cohort of 178 patients with non-clinical APS. Pengo et al. [11] prospectively studied 104 aPL positive patients and obtained more obstetric morbidity among those with positivity for the 3 types of aPL. May Chi Soh et al. [36] retrospectively studied 73 patients with positive aPL without clinical disease and compared them with 73 patients with defined APS and with 292 negative controls for aPL. They concluded that those with defined APS had 4 times more risk of obstetric complications, with no differences between asymptomatic aPL patients and controls. In a prospective study by Mustonen et al. [10], 20% of the 119 patients developed obstetric complications (four of them meeting criteria for APS). Among our patients, only two patients developed obstetric APS. We did not observe any statistically significant association between the antibody profile and the clinical phenotype of these patients, although the results may be limited by the number of patients included.

Regarding the treatment of obstetric APS, although a Cochrane review in 2005 [37] concluded that the management of these patients remained uncertain, the use of ASA, associated or not, with LWMH is generally recommended [38]. The opinion of different authors about the treatment of pregnancy in asymptomatic women with positive aPL is also variable. An observational study by Del Ross et al. [39] reviewed 139 pregnancies in 114 women with aPL who did not meet clinical criteria for APS. There were no significant differences in treatment. However, they concluded that its use could be justified because pregnancy itself and the presence of aPL is additional risk factors for thrombosis. Amengual et al. [40] published a meta-analysis with a review of the literature on primary prophylaxis to prevent obstetric complications in asymptomatic women with aPL. They concluded that there is no sufficient evidence to treat these patients in the absence of other risk factors. In our study, however, we found a protection with prophylactic treatment with ASA (100 mg/day), and in some cases associated with LWMH, in the prevention of early miscarriages. However, two cases of IUGR and one preterm delivery were observed in three of the pregnancies, all in treated patients. These findings could be explained due to early obstetric morbidity prevention with a higher rate of late complications. More prospective studies with larger sample sizes are necessary to corroborate these observations.

In summary, we conclude that smoking, hypertension, thrombocytopenia, and the number of positive aPL are independent risk factors for thrombosis in patients with aPL positive without clinical criteria for APS. In these patients, it seems that ASA has a limited protective role. At the obstetric level, we cannot draw conclusions about the aPL profile, probably because of the limited sample size. However, we observed that prophylactic treatment might be effective in the prevention of early abortions, with a higher rate of live births.

## Supplementary Information

Below is the link to the electronic supplementary material.

Supplementary file1 (DOC 134 KB)

Supplementary file2 (DOCX 22 KB)

Supplementary file3 (DOC 123 KB)

Supplementary file4 (DOCX 22 KB)

Supplementary file5 (DOCX 13 KB)
