# Supplementary figures and images for: Risk Factors for the Development of the Disease in Antiphospholipid Antibodies Carriers: A Long-term Follow-up Study
Source: Clin Rev Allergy Immunol. 2021 Jul 3;62(2):354–62. doi: 10.1007/s12016-021-08862-5 (PMC8994711; doi:10.1007/s12016-021-08862-5)

**Supplementary Figure 1.**


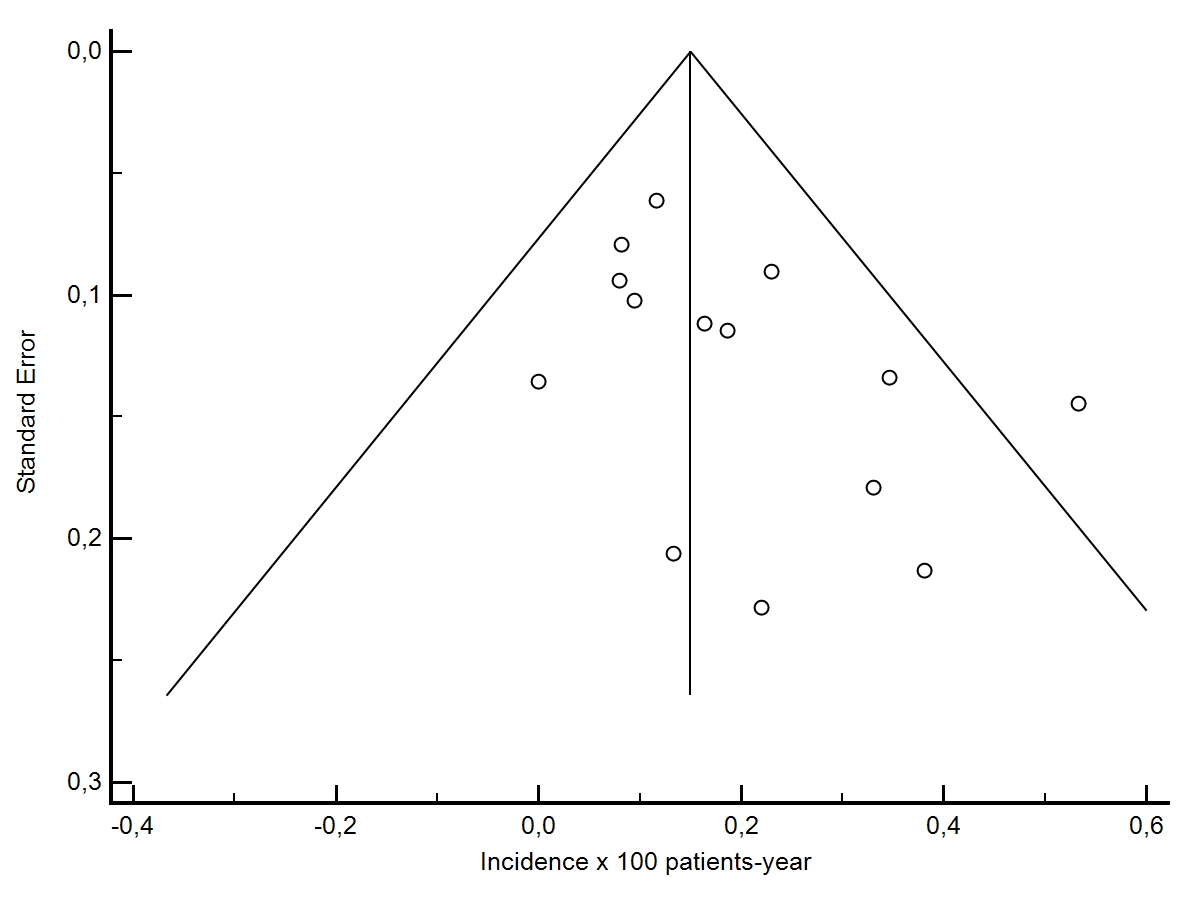


-3

-2

5

3

6

-1

1

0.0

-4

4

2

Supplement: Supplementary file 1 — Supplementary file1 (DOC 134 KB) [file 12016_2021_8862_MOESM1_ESM.doc]
