# Supplementary material for: Risk Factors for the Development of the Disease in Antiphospholipid Antibodies Carriers: A Long-term Follow-up Study
Source: Clin Rev Allergy Immunol. 2021 Jul 3;62(2):354–62. doi: 10.1007/s12016-021-08862-5 (PMC8994711; doi:10.1007/s12016-021-08862-5)
Supplement: Supplementary file 2 — Supplementary file2 (DOCX 22 KB) [file 12016_2021_8862_MOESM2_ESM.docx]

**Supplementary Table 1**.- Thrombosis in patients with positive antiphospholipid antibodies: review of the literature.

|  | **Design** | **Follow-up**  **(years)** | **Nº of**  **patients** | **Age**  **(years)** | **Female**  **(%)** | **SLE**  **%** | **ASA Treatment.**  **(%)** | **Thrombosis rate**  **(100 pat-y)** | **Other results** |
| --- | --- | --- | --- | --- | --- | --- | --- | --- | --- |
| Finazzi (1996)  (**5**) | Prospective | 3.9 | 243 | 39 | 67.2 | 19 | ND | 0.95 | Independent risk factor for thrombosis: previous thrombosis and Ig G aCL |
| Shah (1998)  (**3**) | Retrospective | 10 | 21 | 40 | 90.5 | 100 | 14 | 3.8 | 52% of the aPL carriers developed APS |
| Girón (2004)  (**4**) | Prospective | 1.5 | 178 | 37.4 | 65.7 | 0 | 1.7 | 0.0 | 1. APS Group: 51 fetal loss.  2. Asymptomatic Group: no fetal loss |
| Forastiero (2005)  (**6**) | Prospective | 1.88 | 75 | 42 | 68 | 32 | ? | 3.3 | Independent risk factor for thrombosis: male sex, previous thrombosis AB2GPI (*p*<0.05) |
| Erkan (2007)  (**12**) | Prospective  Group 1 CT.  Group 2 cohort | G 1: 2.3  G 2: 2.5 | G 1: 98  G 2: 74 | G 1: 42.9  G 2: 47.5 | G 1: 89.8  G 2: 95.9 | G 1: 65.3  G 2: 35.1 | G1: 49.0  G2: 82.3 | G 1: 1.33  G 2: 2.2 | G1. ASA does not protect. Low thrombosis rate in asymptomatic aPL+ patients  G2. ASA does not protect |
| Hereng (2008)  (**13**) | Retrospective | 5.3 | 103 | 41.4 | 88.4 | 36 | 72.8 | 3.5 | ASA protects in SLE patients (*p*=0.03) |
| Ruffati (2009)  (**10**) | Retrospective | 4.9 | 370 | 34 | 93 | 35.7 | 37.6 | 1.64 | ASA protects (*p*=0.000)  Independent risk factor for thrombosis: hypertension (*p*=0.000) and IgG aCL (*p*=0.008) |
| Pengo (2011)  (**9**) | Prospective | 4.5 | 104 | 45 | 78.8 | 13 | 35.6 | 5.3 | ASA does not protect.  Independent risk factor for thrombosis: male sex (*p*=0.007) and cardiovascular risk factors (*p*=0.01).  Increased obstetric morbidity in triple aPL+ patients. |
| Ruffati (2011)  (**11**) | Prospective | 1.8 | 258 | 40 | 86.4 | 27 | 54.5 | 1.86 | Independent risk factor for thrombosis: hypertension and LA (*p*<0.05) |
| Mustonen (2014)  (**8**) | Prospective | 9.1 | 119 | 35 | 89 | 37 | 38 | 0.8 | ASA does not protect  Independent risk factor for thrombosis: Smoking |
| Yelnik (2016)  (**14**) | Retrospective | 13 | 98 | 41 | 86.7 | 42.9 | 67.3 | 2.3 | ASA does not protect  Increased thrombosis risk in triple aPL+ |
| Hisada (2017)  (**15**) | Retrospective | 10.7 | 291 | 49 | 82.5 | 41.6 | 30 | 1.16 | aPL carriers with low platelet counts are at high risk of developing thrombosis |
| Demetrio | Retrospective | 12.2 | 138 | 41 | 86.2 | 19.5% SLE | 73.9 | 0.82  Single aPL +: 0.7  Double aPL+: 0,4  Triple aPL+: 3.0 | Independent risk factor for thrombosis:  hypertension, Smoking, thrombocytopenia and triple aPL (*p*<0.005).  ASA protects from early pregnancy loss |

APS: antiphospholipid syndrome; aPL: antiphospholipid antibodies; LA: lupus anticoagulant; aCL: anticardiolipin antibodies; AB2GPI: anti-B2 glycoprotein antibodies; CT: clinical trials; SLE: systemic lupus erythematosus; ND: no dates; pat-y: patients-year; ASA: acetylsalicylic acid; p: stadistical significance. G: group.
