# Supplementary material for: Risk Factors for the Development of the Disease in Antiphospholipid Antibodies Carriers: A Long-term Follow-up Study
Source: Clin Rev Allergy Immunol. 2021 Jul 3;62(2):354–62. doi: 10.1007/s12016-021-08862-5 (PMC8994711; doi:10.1007/s12016-021-08862-5)
Supplement: Supplementary file 3 — Supplementary file3 (DOC 123 KB) [file 12016_2021_8862_MOESM3_ESM.doc]

Supplementary Table 2.- Steps in the method conditional backward, starting from the model 0: tobacco, HBP, dyslipemia, triple positivity and thrombopenia.
Variables in the equation	
	B	Std. Err.	Wald	df	Sig.	OR	95% C.I	
							Lower	Upper	
Step 1		Tobacco	2.327	0.790	8.672	1	0.003	10.249	2.178	48.235	
		HBP	2.168	0.878	6.102	1	0.014	8.741	1.565	48.824	
		Dyslipemia	1.054	0.860	1.502	1	0.220	2.868	0.532	15.472	
		Triple positivity 	0.835	1.078	.600	1	0.438	2.306	0.279	19.083	
		Thrombopenia	1.409	1.000	1.987	1	0.159	4.094	0.577	29.054	
		Constant	-4.539	0.796	32.497	1	0.000	0.011			
Step 2		Tobacco	2.315	0.781	8.778	1	0.003	10.122	2.189	46.801	
		HBP	2.279	0.864	6.952	1	0.008	9.769	1.795	53.168	
		Dyslipemia	1.006	0.843	1.425	1	0.233	2.734	0.524	14.254	
		Thrombopenia	1.838	0.815	5.094	1	0.024	6.286	1.274	31.021	
		Constant	-4.534	0.796	32.476	1	0.000	0.011			
Step 3		Tobacco	2.441	0.769	10.068	1	0.002	11.484	2.543	51.871	
		HBP	2.653	0.804	10.898	1	0.001	14.193	2.938	68.555	
		Thrombopenia	1.999	0.797	6.286	1	0.012	7.379	1.547	35.202	
		Constant	-4.546	0.795	32.676	1	0.000	0.011			
	
HBP: high blood pressure
