# Supplementary material for: Risk Factors for the Development of the Disease in Antiphospholipid Antibodies Carriers: A Long-term Follow-up Study
Source: Clin Rev Allergy Immunol. 2021 Jul 3;62(2):354–62. doi: 10.1007/s12016-021-08862-5 (PMC8994711; doi:10.1007/s12016-021-08862-5)
Supplement: Supplementary file 4 — Supplementary file4 (DOCX 22 KB) [file 12016_2021_8862_MOESM4_ESM.docx]

**Supplementary Table 3.-** Demographic, clinical, analytical and treatment data of aPL carriers who developed thrombosis.

| **Age** | **Sex** | **Follow-up (years)** | **Years to**  **APS** | **CVRF** | **Associated**  **diseases** | **Antibodies** | **Thrombotic**  **event** | **Diagnosis** | **Treatment** |
| --- | --- | --- | --- | --- | --- | --- | --- | --- | --- |
| 38 | Female | 26 | 17 | Smoking | SLE | AntiB2GPI Ig G | DVT | Ultrasound doppler | ASA 100 mg  Hydroxychloroquine  Prednisone  Cyclophosphamide  Acenocumarol (post-thrombosis) |
| 46 | Female | 9 | 8 |  | SLE | aCL IgM  aCL Ig G  AB2GPI IgG | AMI | Electrocardiogram | ASA 100 mg  Hydroxychloroquine |
| 50 | Female | 11 | 8 | Smoking HBC | Chronic pancreatitis | aCL IgM  aCLIgG | AMI | Electrocardiogram | ASA  Clopidogrel  Simvastatin |
| 46 | Male | 15 | 6 | Smoking HBC  DL | Ankylosing spondylitis | aCL IgM  aCL IgG | AMI | Electrocardiogram | ASA 100 mg  Simvastatin  Prednisone |
| 73 | Female | 15 | 9 | HBC | Breast cancer  Undifferentiated connective tissue  Hypothyroidism | aCL IgG | Pulmonary embolism | Computed tomography | ASA 100 mg  Acenocumarol (post-thrombosis) |
| 68 | Female | 10 | 6 | HBC  DL | Sjögren | aCL IgM | Thrombosis central artery of the retina | Fundus examination | Post-thrombosis:  ASA 100 mg  Clopidogrel  Simvastatin |
| 22 | Female | 14 | 4 | Former smoker | No | aCL IgM  AB2GPI IgM  AL | Anterior ischemic optic neuropathy | Fundus examination | ASA 100 mg  Prednisone  Acenocumarol (post-thrombosis) |
| 59 | Female | 14 | 6 | Smoking o  DL | No | aCL IgM  AL | AMI | Electrocardiogram | ASA  Prednisone |
| 29 | Female | 9 | 7 | Smoking | SLE  Asthma  Hypothyroidism | aCL IgM  aCL IgG | Transient ischemic attack | Clinic examination | ASA100mg  Hydroxychloroquine  Methylprednisolone  Azathioprine  Acenocumarol (post-thrombosis) |
| 45 | Male | 16 | 11 | Smoking HBC | No | aCL IgG  aCL IgM  AB2GPI IgG  AL | Acute stroke | Computed tomography | ASA 100 mg  Hydroxychloroquine  Prednisone  Acenocumarol (post-thrombosis) |
| 38 | Male | 8 | 5 months | Smoking | Mutation Leiden V factor | aCL IgM  AB2GPI IgM | Pulmonary embolism | Computed tomography | Prednisone  Gamma globulin  Acenocumarol (post-thrombosis) |
| 80 | Female | 5 | 1 | HBC | Undifferentiated connective tissue | aCL IgM | Anterior ischemic optic neuropathy | Fundus examination | ASA 100 mg  Prednisone |
| 49 | Female | 15 | 9 | HBC  DL | SLE | aCL IgG | Thrombosis central artery of the retina | Fundus examination | Hydroxychloroquine  Prednisone  Methotrexate  Azathioprine  Simvastatin  Acenocumarol (post-thrombosis) |

APS: antiphospholipid syndrome; CVRF: cardiovascular risk factors; HBC: high blood pressure; DL: dyslipidemia OH: enolism; SLE: systemic lupus erythematosus; aCL: anticardiolipin antibody; AB2GPI: anti beta-2 glycoprotein I antibodies; LA: lupus anticoagulant; DVT: deep vein thrombosis; AMI: acute myocardial infarction; ECG: electrocardiogram; ASA: acetylsalicylic acid.
