# Supplementary material for: Risk Factors for the Development of the Disease in Antiphospholipid Antibodies Carriers: A Long-term Follow-up Study
Source: Clin Rev Allergy Immunol. 2021 Jul 3;62(2):354–62. doi: 10.1007/s12016-021-08862-5 (PMC8994711; doi:10.1007/s12016-021-08862-5)
Supplement: Supplementary file 5 — Supplementary file5 (DOCX 13 KB) [file 12016_2021_8862_MOESM5_ESM.docx]

**Supplementary Table 4.-** Description of aPL carriers who developed obstetric antiphospholipid syndrome.

CVRF: cardiovascular risk factors; aCL: anticardiolipin antibodies; ASA: acetylsalicylic acid; LMWH: low molecular weight heparin.

| **Age**  **(yrs)** | **Nº of pregnancy** | **CVRF** | **Associated diseases** | **Antibodies** | **Treatment** | **Obstetric**  **event** |
| --- | --- | --- | --- | --- | --- | --- |
| 31 | 1º | No | Sjögren | aCL IgM | No | Fetal loss  >10 weeks |
| 31 | 3º | No | No | aCL IgM | ASA  + LMWH | Birth  < 34 weeks |
